# Innovative telemedicine and mobile phone technology in the management of diabetic foot ulcers

**DOI:** 10.1186/1757-1146-8-S2-P4

**Published:** 2015-09-22

**Authors:** Damien Clark, Lloyd F Reed, Monika Janda, Petrea L Cornwell, Peter A Lazzarini

**Affiliations:** 1 School of Clinical Sciences, Queensland University of Technology, Kelvin Grove, Qld, 4059, Australia; 2 Department of Podiatry, Metro North Hospital and Health Service, Queensland Health, Chermside, Qld, 4032, Australia; 3 Institute of Health and Biomedical Innovation, Queensland University of Technology, Kelvin Grove, Qld, 4059, Australia; 4 School of Public Health and Social Work, Queensland University of Technology, Kelvin Grove, Qld, 4059, Australia; 5 Allied Health Research Collaborative, Metro North Hospital and Health Service, Queensland Health, Chermside, Qld, 4032, Australia; 6 Behavioural Basis of Health Program, Griffith Health Institute, Griffith University, Mt Gravatt, Qld, 4122, Australia

**Keywords:** Telemedicine, diabetic, foot, ulcer

## Background

Diabetic foot ulcers (DFU) are a leading cause of diabetes-related hospitalisation and can be costly to manage without access to appropriate expert care. Within Queensland and indeed across many parts of Australia, there is an inequality in accessing specialist services for individuals with DFU. Recent National Health and Medical Research Council (NHMRC) diabetic foot guidelines recommend remote expert consultation with digital imaging should be made available to people with DFU to improve their clinical outcomes. Telemedicine appears to show promise in improving access to diabetic foot specialist services; however diabetic foot telemedicine models to date have relied upon videoconferencing, store and forward technology and/or customised appliances to obtain digital imagery which all require either expensive infrastructure or a timed reply to the request for advice. Whilst mobile phone advice services have been used with success in general diabetes management and telehealth services have improved diabetic foot outcomes, the rapid emergence in the use of mobile phones has established a need to review the role that various forms of telemedicine play in the management of DFU. The aim of this paper is to review traditional telemedicine modalities that have been used in the management of DFU and to compare that to new and innovative technology that are emerging.

## Process

Studies investigating the management of DFU using various forms of telemedicine interventions will be included in this review. They include the use of videoconferencing technology, hand held digital still photography purpose built imaging devices and mobile phone imagery. Electronic databases (Pubmed, Medline and CINAHL) will be searched using broad MeSH terms and keywords that cover the intended area of interest.

## Findings

It is anticipated that the results of this narrative review will provide delegates of the 2015 Australasian Podiatry Conference an insight into the types of emerging innovative diagnostic telemedicine technologies in the management of DFU against the backdrop of traditional and evidence based modalities. It is anticipated that the findings will drive further research in the area of mobile phone imagery and innovation in the management of DFU.

